# Endothelial KCa channels: Novel targets to reduce atherosclerosis-driven vascular dysfunction

**DOI:** 10.3389/fphar.2023.1151244

**Published:** 2023-03-31

**Authors:** O. Daniel Vera, Heike Wulff, Andrew P. Braun

**Affiliations:** ^1^ Department of Physiology and Pharmacology, Libin Cardiovascular Institute, Cumming School of Medicine, University of Calgary, Calgary, AB, Canada; ^2^ Department of Pharmacology, School of Medicine, University of California, Davis, CA, United States

**Keywords:** atherosclerosis, endothelium, KCa channel, SKA-31, vascular function

## Abstract

Elevated levels of cholesterol in the blood can induce endothelial dysfunction, a condition characterized by impaired nitric oxide production and decreased vasodilatory capacity. Endothelial dysfunction can promote vascular disease, such as atherosclerosis, where macrophages accumulate in the vascular intima and fatty plaques form that impair normal blood flow in conduit arteries. Current pharmacological strategies to treat atherosclerosis mostly focus on lipid lowering to prevent high levels of plasma cholesterol that induce endothelial dysfunction and atherosclerosis. While this approach is effective for most patients with atherosclerosis, for some, lipid lowering is not enough to reduce their cardiovascular risk factors associated with atherosclerosis (e.g., hypertension, cardiac dysfunction, stroke, etc.). For such patients, additional strategies targeted at reducing endothelial dysfunction may be beneficial. One novel strategy to restore endothelial function and mitigate atherosclerosis risk is to enhance the activity of Ca^2+^-activated K^+^ (KCa) channels in the endothelium with positive gating modulator drugs. Here, we review the mechanism of action of these small molecules and discuss their ability to improve endothelial function. We then explore how this strategy could mitigate endothelial dysfunction in the context of atherosclerosis by examining how KCa modulators can improve cardiovascular function in other settings, such as aging and type 2 diabetes. Finally, we consider questions that will need to be addressed to determine whether KCa channel activation could be used as a long-term add-on to lipid lowering to augment atherosclerosis treatment, particularly in patients where lipid-lowering is not adequate to improve their cardiovascular health.

## Introduction

Cardiovascular diseases (CVDs) are the leading cause of death worldwide and account for approximately 31% of global deaths (World Health Organization, 2021). A major risk factor for CVD is atherosclerosis, a condition that begins when the levels of low-density lipoprotein cholesterol (LDL-C) in the blood exceed the physiological range resulting in fatty plaque formation on the luminal surface of conduit arteries that impedes blood flow (Libby et al., 2019). CVDs associated with atherosclerosis are called atherosclerotic cardiovascular disease (ASCVD) events and commonly include coronary heart disease (CHD), stroke, and peripheral arterial disease (Stone et al., 2014). Atherosclerosis consists of three phases: initiation, progression, and complications (Libby et al., 2019). The mechanisms behind these phases have been extensively studied to understand how atherosclerosis occurs and how these mechanisms can be modified *via* treatments to mitigate atherosclerosis.

Major factors contributing to atherosclerosis initiation include high levels of LDL-C particles circulating in the blood that pass through the luminal endothelial cell (EC) monolayer to enter the intima of the arterial wall (Libby et al., 2019) and the interactions between endothelial cells and hemodynamic shear stress (Davies, 2009; Chiu and Chien, 2011). LDL-C particles can undergo a variety of oxidative modifications induced by reactive oxygen species (ROS), resulting in oxidized LDL-C (oxLDL-C) particles (Hansson and Hermansson, 2011). The oxLDL-C can then interact with ECs to induce endothelial dysfunction, a condition characterized by impaired vasodilatory capacity and an overexpression of cellular adhesion molecules (Hadi et al., 2005; Pirillo et al., 2013), such as intercellular adhesion molecule 1 (ICAM-1) and vascular cell adhesion molecule 1 (VCAM-1) which promote recruitment of circulating monocytes to the endothelium (Cybulsky and Gimbrone, 1991; Nakashima et al., 1998). Furthermore, the endothelium can also bind circulating platelets that express adhesion molecules such as P-selectin to recruit more monocytes to the endothelium (Kuijper et al., 1998; Schulz and Massberg, 2012). The recruited monocytes then extravasate through the endothelium to infiltrate the intimal layer of the vascular wall (Gisterå and Hansson, 2017). Within the intima, factors secreted by the dysfunctional ECs, including macrophage-colony stimulating factor (M-CSF) and granulocyte-macrophage colony-stimulating factor (GM-CSF), induce the monocytes to differentiate into macrophages (Gisterå and Hansson, 2017), which then take up oxLDL-C molecules *via* scavenger receptors, transforming the macrophages into foam cells (Gisterå and Hansson, 2017; Libby et al., 2019). Once present in the intimal layer of the arterial wall, macrophages can also proliferate independently of monocyte extravasation to increase the size of the fatty plaque (Robbins et al., 2013).

Hemodynamic shear stress is an important regulator of endothelial function and is generated by the unidirectional flow of blood along the luminal surface of the endothelium (Davies, 2009; Chiu and Chien, 2011). Mechanotransduction pathways in the endothelium transform this mechanical force into biochemical, signal transduction events that regulate endothelial gene transcription and protein expression, thereby shaping the cellular phenotype of the endothelium (Davis et al., 2023). Decreased shear stress, due to low vs. high blood flow, oscillatory/disturbed vs. laminar blood flow, at focal sites within the arterial tree, such as bifurcations, arterial branches, sites of arterial narrowing (e.g., stenosis) and curvatures, contribute directly to atherogenesis (Chiu and Chien, 2011; Davies et al., 2013; Zhou et al., 2014). This conclusion is supported by the non-randomized pattern of atherosclerotic plaque formation observed *in vivo* (Pedersen et al., 1999; Cheng et al., 2006). Whereas moderate to high laminar shear stress promotes the production of endothelium-derived vaso-protective factors, such as nitric oxide and prostacyclin, prolonged exposure to low or oscillatory blood flow can impair vascular function and health through mis-orientation of endothelial cell patterning, increased endothelial barrier permeability and oxidative stress, and elevated expression of pro-inflammatory genes in the endothelium (Davies et al., 2013; He et al., 2022). High and low shear stress thus have anti- and pro-atherogenic effects, respectively, on the vascular endothelium, and detailed descriptions of the signal transduction pathways underlying these processes are available (Davies et al., 2013; He et al., 2022).

**FIGURE 1:**
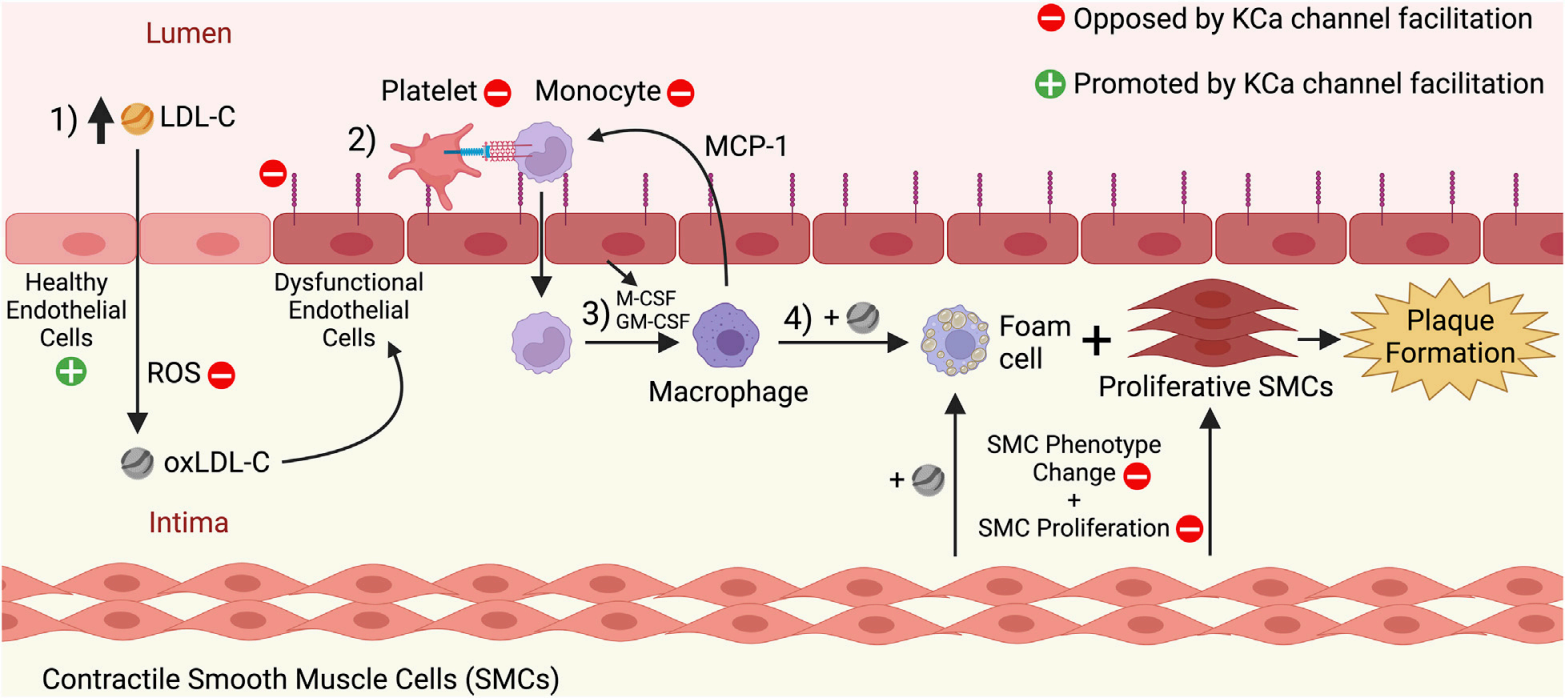
Initiation and progression stages of atherosclerosis, and the effects of KCa channel facilitation on these processes. 1) High levels of circulating low-density lipoprotein cholesterol (LDL-C) in the blood pass through the endothelium and enter the arterial intima. There, the LDL-C undergoes oxidation by reactive oxygen species (ROS), leading to oxidized LDL-C (oxLDL-C). The oxLDL-C can induce dysfunction of the endothelium, which manifests as structural and functional changes to the endothelial cells, such as an increased expression of adhesion molecules like ICAM-1 and VCAM-1. 2) Endothelial dysfunction promotes the recruitment of platelets and monocytes to the endothelium, and the monocytes then extravasate through the endothelium to enter the intima. 3) There, cytokines secreted by the dysfunctional endothelium (e.g., M-CSF, GM-CSF) induce the differentiation of monocytes into macrophages. Macrophages in the intima secrete cytokines such as MCP-1 that recruit more monocytes to the endothelium and thus promote a cyclical process that increases the number of macrophages in the intima. 4) Macrophages take up oxLDL-C *via* scavenger receptors and become foam cells. Concurrently, contractile smooth muscle cells (SMCs) dedifferentiate and become proliferative SMCs and SMC-derived foam cells. The continuing proliferation of foam cells and other SMC-derived cell types contributes to atherosclerotic fatty plaque formation. The red minus signs indicate processes that would be inhibited by KCa channel facilitation and oppose atherosclerosis, including decreasing ROS levels in the intima, inhibiting adhesion molecule expression by the endothelium, inhibiting platelet/monocyte recruitment to the endothelium, and inhibiting SMC phenotype change and proliferation. The green plus sign indicates that healthy endothelial function would be promoted by KCa channel facilitation, and the molecular mechanisms behind this effect are highlighted in Figure 2. This figure was created with www.BioRender.com.

Once initiated, sites of atherosclerosis progress as foam cells accumulate in the intimal region of the arterial wall to form a fatty plaque or atherosclerotic lesion that bulges into the vascular lumen (Libby et al., 2019). Additionally, mature, contractile vascular smooth muscle cells (VSMCs) dedifferentiate and adopt non-contractile, proliferative phenotypes during atherosclerosis progression (Grootaert and Bennett, 2021). Several of these dedifferentiated phenotypes aggravate the plaque, such as the hyperplastic VSMCs that enlarge the fatty plaque (Grootaert and Bennett, 2021) and the foam cell-like VSMCs that take up oxLDL-C particles and promote plaque instability (Allahverdian et al., 2014; Wang et al., 2019). The growing fatty plaque decreases the luminal cross-sectional area, which inhibits proper blood flow through the artery (Libby et al., 2019). The events driving the initiation and progression of atherosclerosis can take decades before CVDs of atherosclerotic origin become apparent (Libby et al., 2019). Additionally, complications can occur when overt atherosclerosis has progressed for several years. One of these complications is plaque rupture, a condition in which the plaque bursts and contents such as tissue factors are released into the vascular lumen, leading to the formation of blood clots that can trigger acute events like myocardial infarction or ischemic stroke (Wilcox et al., 1989; Libby, 2013). The major steps of atherosclerosis initiation and progression are summarized in Figure 1.

Atherosclerosis is also characterized by impaired arterial vasodilatory capacity, a hallmark feature of endothelial dysfunction (Davignon and Ganz, 2004). The endogenous muscarinic receptor agonist acetylcholine (ACh) normally induces vasodilation in healthy arteries by acting on the endothelium, but in early and advanced atherosclerotic arteries from rodents (e.g., aorta), the vasodilatory response to ACh is largely blunted (Crauwels et al., 2003; d'Uscio et al., 2001; Lefer et al., 1987), whereas in humans with atherosclerosis, ACh can evoke constriction in the coronary circulation (Ludmer et al., 1986). The latter observation is often used as a clinical biomarker of endothelial dysfunction (Bairey Merz et al., 2020). In addition to the overexpression of EC adhesion molecules, endothelial dysfunction is also characterized by a decreased bioavailability of the vasodilatory substance nitric oxide (NO) (Davignon and Ganz, 2004; Hadi et al., 2005). NO protects the arterial wall from events that initiate atherosclerosis by inhibiting endothelial expression of adhesion molecules that bind monocytes (De Caterina et al., 1995), opposing the proliferation of VSMCs (Jeremy et al., 1999), preventing platelet activation and aggregation (Riddell and Owen, 1999), and decreasing the levels of ROS that oxidize LDL-C molecules (Violi et al., 1999). Therefore, conditions that increase endothelial NO production may mitigate endothelial dysfunction and reduce the initiation and/or progression of atherosclerosis.

## Current pharmacological treatments for atherosclerosis focus on lipid lowering

Currently, the most common therapeutic treatment for atherosclerosis is a class of drugs called statins (Libby et al., 2019). Statins inhibit the 3-hydroxy-3-methylglutaryl-CoA (HMG-CoA) reductase enzyme which catalyzes the rate-limiting step of LDL-C synthesis in hepatocytes (Endo, 1992). Statin treatment decreases LDL-C levels in the blood and mitigates atherosclerosis development (Libby et al., 2019). For this reason, guidelines such as those from the American College of Cardiology/American Heart Association (Stone et al., 2014; Grundy et al., 2019) and the European Society of Cardiology (Mach et al., 2019) stress the importance of lipid lowering *via* statin drugs for individuals with CVDs or with a high risk of developing CVDs. While statins are effective and safe for most patients with atherosclerotic disease (Collins et al., 2016), these drugs may not be adequate to control atherosclerosis development in some patients. For example, individuals with familial hypercholesterolemia (a condition in which LDL-C endocytosis by the liver is impaired) have very high circulating LDL-C levels even with statin treatment and so they may benefit from an additional treatment to mitigate their elevated CVD risk (Bouhairie and Goldberg, 2015; Libby et al., 2019). For example, a combination treatment consisting of statins alongside other lipid-lowering drugs such as inhibitors of proprotein convertase subtilisin/kexin type 9 (PCSK9) may help patients reach their desired LDL-C levels (Grundy et al., 2019; Lloyd-Jones et al., 2022). Another condition that complicates lipid lowering is statin intolerance, which occurs when patients on statins are unable to tolerate their statin type and/or its dosage and stop taking the medication (Fitchett et al., 2015). Potential side-effects associated with statin therapy include muscle pain or myopathy (the most commonly reported symptom), elevated blood glucose, increased plasma liver enzymes and nephropathy, which can be verified diagnostically through detection of elevated plasma levels of creatine kinase and myoglobin (associated with skeletal muscle damage) or liver transaminases (e.g., AST and ALT) (Ward et al., 2019). While the frequency of such events in statin-treated patients appears to be low (<0.1%) (Newman et al., 2019), patient non-compliance with statin therapy can reach up to 10% (as reported in observational studies) and may reflect expected or perceived harmful effects of the drugs (i.e., nocebo effect) (Wood et al., 2020; Herrett et al., 2021). To mitigate side-effects, some statin intolerant patients may benefit from using a non-statin drug (e.g., ezetimibe to reduce cholesterol absorption in the GI tract) in addition to low-dose statin treatment that would decrease detectable tissue damage (Fitchett et al., 2015). This unmet clinical need thus represents an opportunity to identify novel therapeutic strategies that either could substitute for statins or be combined with conventional statin therapy.

Although lipid lowering is a crucial strategy for mitigating atherosclerosis development (Stone et al., 2014; Grundy et al., 2019; Mach et al., 2019), it should also be noted that high LDL-C levels induce endothelial damage that promotes atherosclerosis (Hadi et al., 2005). For this reason, exploring mechanisms that mitigate endothelial dysfunction in atherosclerosis would also prove beneficial. Earlier studies have shown that statins can decrease CVD risk by improving endothelial function, independent of their lipid-lowering capability (Wolfrum et al., 2003; Ii and Losordo, 2007). This pleiotropic action implies that it may be advantageous to explore additional strategies that improve endothelial function to mitigate atherosclerosis. Recent studies from our group have shown that pharmacological enhancement of endothelial Ca^2+^-activated K^+^ (KCa) channel activity reverses endothelial dysfunction in the settings of aging (John et al., 2020) and type 2 diabetes (Mishra et al., 2021), raising the possibility that a similar strategy may promote mechanisms that restore endothelial function in atherosclerotic arteries.

## KCa channels: A new target for atherosclerosis mitigation?

Work on Ca^2+^-activated K^+^ (KCa) channels began in the late 1950s when G. Gardos showed that the K^+^ efflux underlying volume regulation in erythrocytes required external Ca^2+^ influx (Gardos, 1958). Since then, three main families of KCa channels have been discovered and their characteristics thoroughly analyzed. KCa channels were originally classified based on their single channel conductance: small-conductance (SK_Ca_, ∼4–14 pS), intermediate-conductance (IK_Ca_, ∼20–80 pS), and big-conductance (BK_Ca_, ∼250 pS) (Vergara et al., 1998). Within each of these three main families exist channel subtypes that are encoded by separate genes. The SK_Ca_ family contains three subtypes (KCa2.1, 2.2, and 2.3), and the IK_Ca_ family contains only a single member (KCa3.1), as does the BK_Ca_ channel family (KCa1.1) (Wei et al., 2005; Salkoff et al., 2006; Kaczmarek et al., 2017; Kshatri et al., 2018). The KCa2.X and KCa3.1 channels are often grouped together because they share considerable primary sequence similarity, but are distantly related to the KCa1.1 channel (Wei et al., 2005). The structure and function of these channels reflect this lineage. For example, the KCa2.X and KCa3.1 channels are voltage independent (Hirschberg et al., 1999; Kshatri et al., 2018), and bind calcium *via* a C-terminal-bound calmodulin (CaM) subunit, which induces the conformational change necessary to open the K^+^ channel pore (Xia et al., 1998; Fanger et al., 1999; Lee and MacKinnon, 2018). On the other hand, KCa1.1 channels are activated by changes in either membrane voltage or by direct C-terminal Ca^2+^ binding, without the need for calmodulin (Horrigan and Aldrich, 2002; Latorre et al., 2017). KCa channels are expressed in a variety of cell types. Both KCa2.3 and KCa3.1 are prominently expressed in the vascular endothelium in humans as well as other animals (Köhler et al., 2016). KCa3.1 channels are also expressed in hematopoietic stem cell (HSC)-derived cells including macrophages, erythrocytes, T lymphocytes, and dedifferentiated, proliferative smooth muscle cells (Schmid-Antomarchi et al., 1997; Neylon et al., 1999; Ghanshani et al., 2000; Wulff and Castle, 2010; Feske et al., 2015). In addition to vascular endothelium, KCa2.X channels are found in the brain and cardiac atria (Köhler et al., 1996; Xu et al., 2003). KCa1.1 channel expression is readily observed in many smooth muscle-containing tissues (e.g., vasculature, GI tract, lungs, bladder, uterus, etc.), neurons, and the kidneys, with lower levels in skeletal muscle and endocrine and exocrine glands (Chang et al., 1997; Contreras et al., 2013).

KCa channels have received attention because modifying their activity may help to mitigate some CVDs including atherosclerosis (John et al., 2018). For instance, systemic administration of a pharmacological blocker of KCa3.1 channels resulted in decreased aortic fatty plaque formation and increased fatty plaque stability in atherosclerotic mice (Toyama et al., 2008; Xu et al., 2017; Tharp and Bowles, 2021; Jiang et al., 2022). Conversely, it has also been shown that enhancing the activity of KCa2.X and KCa3.1 channels improved endothelial function in rodent models of aging (John et al., 2020) and type 2 diabetes (Mishra et al., 2021), suggesting that the same may occur in atherosclerotic endothelial dysfunction. The potential benefits of strategies that either inhibit or enhance KCa channel activity at an integrative level are discussed below.

## KCa channel inhibition for atherosclerosis mitigation

Previous studies have reported that KCa3.1 channel blockers can inhibit VSMC proliferation (Köhler et al., 2003) and ROS production by macrophages (Schmid-Antomarchi et al., 1997). Since both VSMC proliferation and ROS production promote atherosclerosis (Libby et al., 2019), blocking KCa3.1 channels in macrophages and VSMCs could be a way to decrease fatty plaque burden in atherosclerosis. Toyama and others (2008) tested this idea by administering TRAM-34, a selective KCa3.1 channel blocker, to Apoe−/− atherosclerotic mice. After treating the mice over 12 weeks daily with TRAM-34, the authors found a significant decrease in fatty plaque area throughout the aorta, especially in the aortic arch, thoracic aorta, and abdominal aorta. Additionally, the authors showed a significant decrease in macrophage-positive area and VSMC-positive area in the plaques of treated mice compared to control, both indications that the plaque was less severe due to a decrease in these cell numbers (Toyama et al., 2008). There was also a reduction in T cell infiltration into the plaques. Since blocking KCa3.1 channels may conceivably decrease the number and/or function of immune cells such as macrophages and T cells, the authors also tested whether KCa3.1 inhibition with TRAM-34 would delay clearance of influenza virus in rats. TRAM-34 at a therapeutic dose did not reduce viral clearance, while the positive control dexamethasone significantly prolonged viral infections. This result, in conjunction with a normal immune cell count in the spleen and thymus, suggests that systemic KCa3.1 channel blockade may not have major adverse effects on immune system function (Toyama et al., 2008). Finally, to better characterize the makeup of the macrophages present in plaques, Xu and others (2017) determined whether KCa3.1 channel blockade impacts the expression of M1 or M2 markers in plaque macrophages. Based on a characterization that has been widely criticized as overly simplistic (Murray et al., 2014; Nahrendorf and Swirski, 2016), but which is useful for therapeutic hypothesis generation, “M1-like” macrophages are pro-inflammatory, whereas “M2-like” macrophages are assumed to display mostly anti-inflammatory properties and promote wound healing (Murray and Wynn, 2011). Xu and others (2017) found that TRAM-34 treatment promoted the polarization of plaque macrophages towards the more anti-inflammatory M2 phenotype, suggesting that systemic administration of a KCa3.1 channel inhibitor can also decrease the number of macrophages that induce inflammation and worsen atherosclerosis.

Although these results indicate that KCa3.1 channel inhibition may help to decrease atherosclerotic plaque formation, some outstanding questions remain. For instance, since KCa3.1 channels are expressed in both innate and adaptive immune cells (Wulff and Castle, 2010; Feske et al., 2015), there may be a risk that this inhibitory strategy could impair the optimal functioning of the immune system, especially with long-term KCa3.1 channel blockade. However, clinical trials examining administration of the KCa3.1 channel blocker senicapoc (ICA-17043) to patients with sickle cell disease for 1 year reported a small increase in the incidence of urinary tract infections, but otherwise gave no indication of clinically significant immunosuppression (Ataga et al., 2011). Another potential concern is whether KCa3.1 channel inhibition may have adverse effects on cell types other than immune cells or VSMCs. Since KCa3.1 channels are also expressed in the vascular endothelium and contribute to vasodilatory signaling (Köhler et al., 2016), a logical question that arises is whether these endothelial channels are blocked too, and if so, whether this blocking would adversely affect endothelial function. Toyama and others (2008) did not directly test endothelial function after TRAM-34 treatment, and so the possibility for impaired endothelial function cannot be ruled out. TRAM-34 itself cannot be tested in humans because of its poor oral bioavailability (Al-Ghananeem et al., 2010) and lack of an Investigational New Drug Application (IND) from the US Food and Drug Administration allowing administration to humans. However, it should be possible to conduct studies in patients with atherosclerosis using senicapoc (ICA-17043), another potent KCa3.1 channel blocker with a half-life of ∼12 days in humans (Ataga et al., 2006). Senicapoc has been tested successfully in Phase I, II, and III clinical trials for sickle cell disease and has exhibited a remarkable safety profile (Ataga et al., 2006; 2011; 2008). While KCa3.1 channel blockade may inhibit detrimental macrophage and VSMC functions that contribute to atherosclerosis, additional studies are warranted to examine whether KCa3.1 channel inhibitors can mitigate atherosclerosis without adversely affecting endothelial function.

## Enhancement of KCa channel activity for atherosclerosis mitigation

**FIGURE 2:**
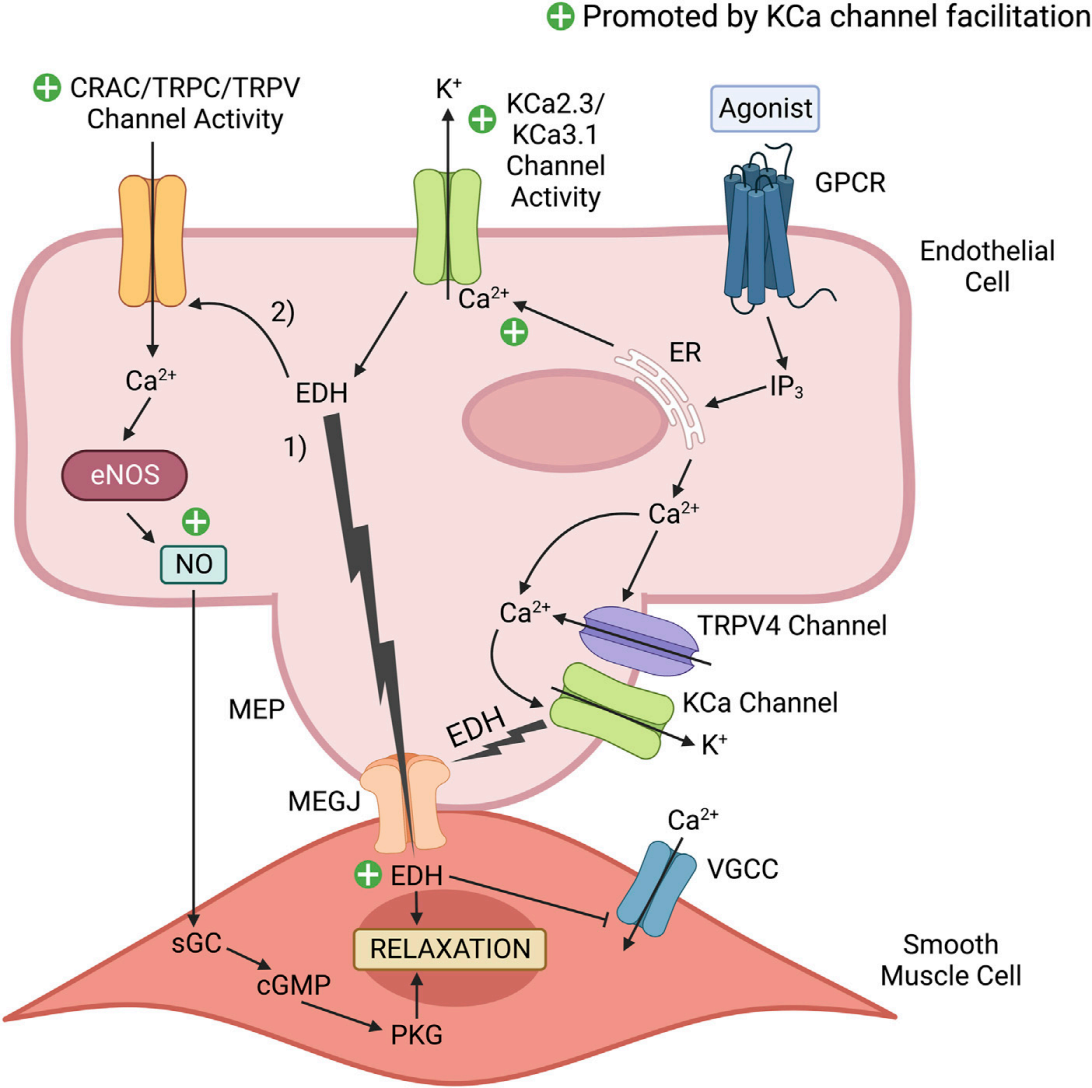
KCa channel positive modulators promote endothelium-dependent vasodilatory signaling. Vasodilatory agonists in the vascular lumen, such as acetylcholine (ACh) bind to their cognate endothelial G-protein coupled receptor (GPCR), which initiates a series of cascading events that results in Ca^2+^ release from the endoplasmic reticulum (ER). Ca^2+^ released from the ER can activate endothelial nitric oxide synthase (eNOS) to produce the potent vasodilator nitric oxide (NO). Additionally, the released Ca^2+^ can activate Ca^2+^-activated K^+^ (KCa) channels that induce endothelium-derived hyperpolarization (EDH). 1) This electrical signal transfers to the smooth muscle cells *via* myoendothelial gap junctions (MEGJs) located within a myoendothelial projection (MEP) to inhibit Ca^2+^ entry *via* voltage-gated calcium channels (VGCCs). Reduced Ca^2+^ entry into the smooth muscle cell limits myosin phosphorylation by Ca^2+^-dependent myosin light chain kinase (MLCK), and the subsequent level of muscle contraction. 2) In addition to EDH-induced vasorelaxation, EDH increases the electrical driving force to promote external Ca^2+^ entry into the endothelial cell *via* Ca^2+^ permeable channels activated in response to a vasodilatory stimulus (e.g., Ca^2+^-release activated Ca^2+^ (CRAC) channels, TRPC3, TRPC4, TRPC6, TRPV1 and TRPV4 channels present in peripheral arteries, such as the aorta). This elevation of Ca^2+^ in the cytosol and beneath the plasma membrane can promote Ca^2+^-dependent vasodilatory signaling, such as NO production by eNOS and KCa channel activity. NO readily diffuses to the adjacent smooth muscle to induce relaxation *via* the cGMP/Protein Kinase G (PKG) pathway. A selective KCa2.X/KCa3.1 positive modulator (e.g., SKA-31) can facilitate the activity of endothelial KCa2.X/KCa3.1 channels, which would enhance EDH and NO production to promote vascular relaxation and healthy endothelial function. The green plus signs highlight mechanisms contributing to endothelium-dependent vasodilation that would be enhanced by KCa channel facilitation. This figure was adapted from CM John et al. (2018) and created with www.BioRender.com.

As previously stated, proper endothelial function is necessary for maintaining vasodilation and good vascular health. The endothelium contributes to vasodilation *via* two important mechanisms: 1) the nitric oxide (NO) pathway and 2) the endothelium-derived hyperpolarization (EDH) pathway (Shimokawa and Godo, 2016). Vasodilatory agonists such as acetylcholine (ACh) and bradykinin (BK) stimulate endothelial G-protein-coupled receptors (GPCRs) complexed with Gαq, leading to the generation of IP_3_ that promotes Ca^2+^ release from the endoplasmic reticulum (ER). This released Ca^2+^ can then activate CaM-bound endothelial nitric oxide synthase (eNOS) to produce NO that will diffuse to adjacent VSMCs to activate guanylyl cyclase and induce vasodilation (Davignon and Ganz, 2004). The Ca^2+^ released from the ER can also activate CaM bound to the C-terminal domain of KCa2.X/KCa3.1 channels to induce their pore opening and subsequent K^+^ efflux (Köhler et al., 2016; Vanhoutte et al., 2017). KCa3.1 channels present within myoendothelial projections at the interface of the endothelium and smooth muscle layer are also activated by Ca^2+^ influx through co-localized TRPV4 channels, which themselves are Ca^2+^-sensitive and can be stimulated by IP_3_-mediated ER Ca^2+^ release, leading to amplified KCa3.1 channel activation (Chen and Sonkusare, 2020). The K^+^ efflux produced by this collective KCa channel activity results in endothelium-derived hyperpolarization (EDH), an electrical signal that transfers electrotonically to VSMCs *via* myoendothelial gap junctions (MEGJs) to induce hyperpolarization, decreased opening of voltage-gated Ca^2+^ channels and a reduction in the level of intracellular Ca^2+^ for VSMC contraction (Garland et al., 2011; Billaud et al., 2014; Jackson, 2022). In large conduit arteries such as the aorta, vasodilation is mostly NO driven, whereas in the smaller resistance arteries, vasodilation is more dependent upon EDH (Shimokawa and Godo, 2016). The NO and EDH mechanisms of endothelial-derived vasodilation are summarized in Figure 2.

It is well documented that vessels exhibiting endothelial dysfunction produce insufficient amounts of NO (Hadi et al., 2005), and so a novel way to mitigate atherosclerosis could be to promote NO production by enhancing endothelial KCa channel activity (see Figure 2). Positive KCa2.X/KCa3.1 channel gating modulator drugs, such as NS309, enhance the stability of the channel’s open conformation that is induced by Ca^2+^-CaM binding (Brown et al., 2017). It is important to clarify that these positive-gating modulator drugs do not activate the KCa channels *per se*; rather, they stabilize the open conformation induced by Ca^2+^-CaM binding. From a biophysical perspective, these positive modulators shift the concentration-response curve for KCa2.X/KCa3.1 channel opening by cytosolic Ca^2+^ in the leftward direction, such that the EC_50_ for cytosolic Ca^2+^ becomes progressively lower with increasing amounts of the positive modulator (Köhler et al., 2016). Thus, positive-gating modulator drugs sensitize KCa channels to Ca^2+^ such that a greater electrical response will be generated at a given concentration of cytosolic Ca^2+^. Experimentally, positive modulators of KCa2.X/KCa3.1 channels evoke concentration-dependent dilation of myogenically active resistance arteries (Sheng et al., 2009; Mishra et al., 2015), implying that these compounds “facilitate” endothelial KCa channel opening under physiologically relevant levels of cytosolic free Ca^2+^. Enhanced KCa channel activity and resulting endothelial hyperpolarization would then augment endothelium-dependent vasodilation by promoting the cellular events underlying this process; 1) external Ca^2+^ entry may be increased by the greater electrical driving force, thereby supporting downstream vasodilatory signaling, 2) augmented Ca^2+^ mobilization will stimulate Ca^2+^-dependent eNOS activity to increase NO biosynthesis, and 3) enhanced endothelial KCa channel activity will increase the direct transfer of hyperpolarizing electrical signals to the adjacent smooth muscle. When administered at a low or threshold concentration, a KCa channel positive modulator would be expected to have minimal effect on cell function under a basal or resting level of cytosolic Ca^2+^, but could substantially augment endothelial KCa channel activity in response to the cytosolic Ca^2+^ elevation evoked by a primary stimulus (e.g., vasodilatory agonist, shear stress, etc.). It may therefore be appropriate to think of these compounds as “facilitator drugs”.

The use of positive ion channel modulators as therapeutic agents is already established and one of the best studied examples is that of benzodiazepines (BZs), a class of drugs used clinically in the treatment of anxiety, panic attacks, insomnia, and as pre-anesthetic agents (Edinoff et al., 2021). BZs bind to the α/γ subunit interface of ionotropic GABA_A_ receptors in the central nervous system and allosterically facilitate channel opening/Cl^−^ influx in response to local GABA release (Sigel and Ernst, 2018). At low, therapeutic concentrations, BZs act as positive allosteric modulators to augment naturally occurring, inhibitory neurotransmission in the brain that acts to dampen excitatory neural pathways (Macdonald and Olsen, 1994; Olsen, 2018). Thus, BZs provide a clear precedent and rationale for how agonistic agents that facilitate the activity of key molecules can be used to strengthen intrinsic signaling pathways and boost functional outcomes. The therapeutic success of BZs and their mechanism of action suggests that drug-induced facilitation of Ca^2+^-dependent KCa channel activity in the vascular endothelium could also be beneficial in the appropriate setting. However, the similarity in mechanism of action to BZs also raises the question of whether there is any type of activity or drug induced downregulation of KCa channels. While BZs are highly effective in treating anxiety, the phenomenon of “benzodiazepine resistance” occurring after prolonged seizure activity is widely known in the epilepsy field and is typically explained by internalization of synaptic GABA_A_ receptors (Vinkers and Olivier, 2012). BZs are therefore only used for acute seizure termination but not for the prevention of seizures. Whereas it might be possible that a similar tolerance develops against KCa channel activators, we have not observed such a phenomenon in the vasculature. In small mesenteric arteries isolated from aged male rats treated daily with the KCa channel activator SKA-31 for 8 weeks, endothelium-dependent vasodilation evoked by either acetylcholine or bradykinin was modestly enhanced vs. vehicle-treated animals (John et al., 2020). In parallel, KCa2.3 and KCa3.1 channel protein expression in mesenteric arteries was not downregulated compared with aged control rats, but was in fact elevated to the levels observed in young adult rats (John et al., 2020). Collectively, these observations suggest that prolonged treatment with a low dosage of the KCa channel activator SKA-31 does not induce tolerance-like effects in the mesenteric vasculature of aged rodents.

In initial proof-of-concept studies, our group showed that positive modulators of endothelial KCa channel activity upregulate both the NO and EDH pathways of vasodilation. Using cultured human umbilical vein endothelial cells, we observed that acute treatment (1–3 min) with NS309 and DCEBIO (KCa2.X/KCa3.1 channel positive-gating modulators) increased agonist-evoked EDH, thereby boosting the electrical driving force to facilitate Ca^2+^ entry into the endothelial cell (Sheng et al., 2009). The likely influx pathways contributing to this Ca^2+^ entry include Ca^2+^-release activated Ca^2+^ (CRAC) channels (Abdullaev et al., 2008; Ruhle and Trebak, 2013), along with various types of transient receptor potential (TRP) cation channels expressed in the endothelium of different vascular beds, such as TRPC3, TRPC4, TRPC6, TRPV1, TRPV3, TRPV4 and TRPA1 (Earley and Brayden, 2015; Jackson, 2022). This Ca^2+^ influx would be expected to support activation of eNOS to generate NO, which enhances endothelial function (Lin et al., 2000; Sheng et al., 2009). Based on such observations, we and others have speculated that facilitation of endothelial KCa channel activity will improve endothelial function (Kerr et al., 2012; Khaddaj-Mallat et al., 2017), and conceivably mitigate the development and/or severity of atherosclerosis.

While the proposed mechanism is intriguing (Figure 2), NS309 and DCEBIO are not well suited for *in vivo* studies, as both compounds may have off-target effects and short plasma half-lives (Köhler et al., 2016). To address these issues, Wulff and others synthesized a novel series of KCa2.X/KCa3.1 channel positive modulators, including SKA-31, using the neuroprotective agent riluzole as a chemical scaffold, with the goal of generating compounds that were more selective and better suited for *in vivo* use (Sankaranarayanan et al., 2009). Unlike previous KCa2.X/KCa3.1 positive-gating modulators, SKA-31 exhibits an EC_50_ of ∼0.3 μM and ∼2 μM for KCa3.1 and KCa2.3 channels respectively, has a plasma half-life of ∼12 h in mice and rats, and has fewer off-target effects and is more selective for KCa2.X/KCa3.1 than other small molecule channel modulators (Sankaranarayanan et al., 2009).

Results from studies discussed above suggest that KCa channel positive gating modulators, such as SKA-31, may be able to mitigate atherosclerosis *via* endothelium-dependent mechanisms. Our group has shown that SKA-31 increases blood flow in the rat coronary circulation (Mishra et al., 2013), inhibits myogenic tone in the cremaster skeletal muscle and cerebral arteries of rats (Mishra et al., 2015), and increases peripheral arterial conductance in the pig (Mishra et al., 2016). Proper endothelial function is necessary for sufficient vasodilation (Sandoo et al., 2010), and so the vasoactive effects that we and other investigators have reported for endothelial KCa channel modulators suggest that facilitation of KCa2.X/KCa3.1 channel activity may promote endothelial function. We have also observed that SKA-31 can restore endothelial function in situations where endothelial dysfunction is present. For instance, we have shown that SKA-31 administration for 8 weeks improved endothelium-dependent vasodilation in the mesenteric arteries of aged rats (i.e., ∼20 months old) to a level similar to that observed in young rats (John et al., 2020). This long-term administration of SKA-31 also modestly increased the protein expression of KCa2.3/KCa3.1 channels along with type 1 IP3 receptor and SERCA2 in small mesenteric arteries from aged male rats (John et al., 2020). Additionally, we have observed that acute treatment with a low concentration of SKA-31 (i.e., 0.3 μM) significantly improved the endothelium-dependent vasodilatory responses to acetylcholine and bradykinin in myogenically active cremaster skeletal muscle resistance arteries from a rat model of spontaneous type 2 diabetes (T2D), and also in isolated intra-thoracic resistance arteries from human T2D subjects (Mishra et al., 2021). This latter result is particularly interesting, as these observations indicate that human arteries remain sensitive to KCa channel facilitation by a positive modulator even after many years of T2D diagnosis and management. Acute SKA-31 treatment also enhanced endothelium-dependent vasodilation in arteries from older, non-T2D subjects (i.e., mean age of 71 years) (Mishra et al., 2021), and since aging is known to promote endothelial dysfunction (Donato et al., 2018; Ungvari et al., 2018), aged human resistance arteries responded to KCa channel facilitation similarly to what we previously reported in aged rat arteries (John et al., 2020).

In addition to their endothelium-dependent mechanisms, KCa channel positive modulators may also have the potential to mitigate atherosclerosis in an endothelium-independent manner. As reported by Bi and others (2013), treatment of serum-starved and quiescent human coronary VMSCs in culture with SKA-31 or other KCa channel activator compounds (e.g., EBIO, NS309) unexpectedly inhibited the transition of these cells from a contractile to a dedifferentiated phenotype and decreased their proliferation in response to mitogenic stimulation by PDGF. At the cellular level, KCa channel activators reduced the expression of KCa3.1 channels, PDGF *β*-receptors, CREB phosphorylation, c-Fos and cell cycle regulatory cyclins in VSMCs (Bi et al., 2013). These observed changes were accompanied by a reduction in KCa3.1 mRNA levels and KCa3.1 macroscopic conductance, as measured by whole-cell patch-clamp, demonstrating reduced KCa3.1 channel activity. In parallel experiments, pharmacological inhibition of VSMC KCa3.1 activity by TRAM-34 or knock-down of KCa3.1 channel expression by siRNA treatment prevented PDGF-stimulated VSMC dedifferentiation and proliferation, and also decreased KCa3.1 mRNA and whole cell current density. These latter effects resulting from KCa3.1 channel inhibition align with observations described in earlier studies using isolated VSMCs and arterial tissue (Köhler et al., 2003; Toyama et al., 2008). Although it seems somewhat paradoxical that two opposing classes of KCa channel compounds (i.e., KCa3.1 channel activators and inhibitors) can produce similar suppression of VSMC proliferation, these provocative data highlight the potential for differential responses of vascular smooth muscle vs. endothelium to KCa channel activators. It is tempting to speculate that the observed effects of KCa channel activators on VSMCs during phenotypic switching may reflect different responses of membrane potential and Ca^2+^ signaling pathways to KCa activators in VSMCs vs. endothelial cells. Going forward, it will be important to discern how KCa channel inhibitors and activators act on VSMCs in the medial layer of arteries once growth/proliferation is triggered. Finally, in addition to SKA-31’s proposed anti-atherogenic effect by inhibiting VSMC dedifferentiation and proliferation, SKA-31-mediated facilitation of platelet KCa3.1 channels has recently been shown to decrease platelet aggregation and adhesion (Back et al., 2022), and such anti-thrombotic actions would also be anticipated to oppose atherogenesis.

Since, as mentioned above, KCa3.1 channel inhibition has been shown to decrease atherosclerosis development in Apoe−/− mice, KCa3.1 blockers and KCa3.1 activators ideally should be compared side-by-side in the same animal experiment and in the same environment in order to determine whether both approaches indeed have therapeutic benefits and whether one approach is superior to the other. Based on the reported observations, we predict that KCa channel activators will prove effective in the reduction of atherosclerosis-associated vascular dysfunction.

Studies thus far suggest that facilitation of KCa2.X/KCa3.1 activity *in vivo* by a positive modulator is free from major adverse effects, but more work will be necessary to determine whether this strategy could have unwanted effects in the setting of atherosclerosis. One concern with the *in vivo* use of KCa channel positive modulators is the possible modification of immune cell numbers and/or activation. KCa3.1 channels are expressed in immune cells such as T lymphocytes, B cells, and macrophages (Feske et al., 2015), and for this reason, our group investigated whether prolonged SKA-31 administration modified T cell numbers and their products, including pro-inflammatory cytokines (John et al., 2020). We found that SKA-31 treatment for 8 weeks in aged rats did not significantly change several sub-populations of adaptive immune cells including CD4^+^ and CD8^+^ T cells, B cells, and NK cells when compared with vehicle-treated aged rats (John et al., 2020). We also found that prolonged drug administration did not significantly change the levels of pro-inflammatory cytokines such as interleukin (IL)-1β, IL-6, IL-18 and tumor necrosis factor-⍺ (TNF-⍺) beyond the levels seen in vehicle-treated, aged controls (John et al., 2020). While these results suggest that facilitation of vascular KCa2.X/KCa3.1 activity may not have major adverse effects on adaptive immunity, it remains unknown whether macrophage number and activity might be impacted by this strategy. Since macrophages proliferate within atherosclerotic plaque lesions (Robbins et al., 2013), it would be useful to determine whether KCa channel positive modulators affect macrophage activity and/or proliferation.

Could the opposite pharmacological actions of KCa channel blockers vs. positive modulators present possible drawbacks in their use as anti-atherogenic agents? Pharmacologically, a non-use-dependent KCa channel blocker (e.g., TRAM-34) would be expected to inhibit both the stimulated and basal activity of KCa3.1, which may suppress target cell function below a baseline level. Such an effect would be analogous to the actions of a local anesthetic agent (e.g., lidocaine) to decrease both stimulated and basal sensory nerve activity below a physiological minimum. On the other hand, application of a KCa channel positive modulator (e.g., SKA-31) to boost the vascular response to a dilatory stimulus would ideally not modify basal endothelial function, as facilitation of endothelial KCa channel activity would not occur under the resting level of cytosolic Ca^2+^.

Another possible concern with KCa channel positive modulators is that they may impact cardiac parameters such as heart rate, since atrial cardiomyocytes in some species (e.g., mice) express KCa2.X channels (Xu et al., 2003; Tuteja et al., 2005) and their facilitation may alter electrical conduction in the atria or promote arrhythmogenesis (Schmitt et al., 2014). Along this line, studies by our group and others suggest that facilitation of KCa channel activity, at least by SKA-31, may not have major adverse effects on the heart. Firstly, KCa3.1 channels do not appear to contribute to arrhythmogenesis in the healthy heart (Schmitt et al., 2014), although one report has suggested a role for KCa3.1 channel current in cardiac pacemaker activity in a transgenic mouse model of catecholaminergic polymorphic ventricular tachycardia (CPVT) (Haron-Khun et al., 2017). These observations imply that positive modulators preferentially acting on KCa3.1 channels (e.g., SKA-31) may not strongly affect KCa2.X channels in atrial myocytes at low doses, or alter cardiac rhythmicity in the absence of arrhythmogenic conditions, such as CPVT. This prediction is supported in part by work from Radtke et al. (2013) who found that acute SKA-31 administration in wild-type mice induced a significant decrease in heart rate only at a high dosage of 100 mg/kg. In addition, we have shown that 8 weeks of daily SKA-31 administration (10 mg/kg) to aged rats did not significantly change their heart rate compared to age-matched untreated controls, but did significantly improve left ventricular ejection fraction and fractional shortening (John et al., 2020). Altogether, these findings suggest that prolonged administration of a KCa channel positive modulator at a low dosage does not have major adverse cardiac effects and is well tolerated. Testing these cardiac parameters in the context of atherosclerosis would be an important next step.

Finally, while SKA-31 has proven very useful as a prototype compound to explore endothelial KCa channel facilitation as a viable strategy to counteract endothelial dysfunction, it is the concept and not the compound that should be emphasized. Once fully validated, this concept will likely promote the development of new KCa channel positive modulators that exhibit the desired potency, selectivity, pharmacokinetics, and safety profiles. These new compounds ideally should have a different pharmacophore than the existing compounds so that they can be patent protected and developed as drugs for human use. Until then, it appears that SKA-31 may be the most suitable pharmacological compound/tool for studying KCa2.X/KCa3.1 channels *in vivo* (John et al., 2018).

## Conclusion

Given its prevalence and the many harmful and debilitating CVDs linked to atherosclerosis, there continues to be a need to develop new and innovative strategies to lessen its burden on human health. While such new strategies are unlikely to replace lipid lowering *via* statin drugs, given their impressive therapeutic effectiveness and safety profile (Collins et al., 2016), they may provide additional treatment benefit through their complementary anti-atherosclerotic actions. As such, strategies designed to enhance endothelial KCa channel activity could be used as an additional treatment option for patients in which statin therapy alone is insufficient to control their atherosclerosis [e.g., some statin intolerant patients (Fitchett et al., 2015)]. If pharmacological facilitation of KCa channel activity is shown to be a safe and effective strategy to prevent and/or treat atherosclerosis, then future studies should explore whether it conveys additional benefits when given alongside statin treatments. If so, facilitation of endothelial KCa channel activity could be developed as a viable add-on to lipid lowering strategies to mitigate atherosclerosis and its associated morbidity and mortality.
